# Results from the IceGut study: tracking the gut microbiome development from mothers and infants up to five years of age

**DOI:** 10.1128/msphere.00745-25

**Published:** 2025-12-02

**Authors:** Agnes Thora Arnadottir, Sigurlaug Skirnisdottir, Stephen Knobloch, Karla F. Corral-Jara, Alexandra Maria Klonowski, Ingibjorg Gunnarsdottir, Viggo Thor Marteinsson

**Affiliations:** 1 Matís ohf. 117046 , Reykjavík, Iceland; 2 Faculty of Food Sciences and Nutrition and Health Science Institute, University of Iceland 63541, Reykjavik, Iceland; 3 Department of Food Technology, Fulda University of Applied Sciences 38883, Fulda, Germany; 4 Unit for Nutrition Research, Landspitali University Hospital 63541, Reykjavik, Iceland; University of California Davis, Davis, California, USA

**Keywords:** 16S rRNA, maternal gut microbiome, infant gut microbiome, child gut microbiome, gut microbiome development, Iceland, gestational diabetes mellitus

## Abstract

The infant gut microbiome plays a critical role in the early development of the immune system, brain function, metabolism, and defense against pathogens. However, data from underrepresented populations, like Iceland, with its distinct dietary and lifestyle habits, remain limited. This paper presents the initial findings from the Icelandic Diet and the Infant Gut Microbiome Development (IceGut) study. Fecal samples were collected at multiple time points, representing 328 unique study identifiers, with one to five samples per child, from before the introduction of solid foods up to 5 years of age, and postpartum samples from 214 mothers. Microbial composition and predicted functional potential were assessed using 16S rRNA gene sequencing. Children in the cohort followed typical gut microbiome maturation, but at 1 year of age, they showed a notably higher relative abundance of *Blautia* than reported in comparable cohorts. This time point marked a transition in both taxonomic composition and predicted functional gene counts. By 5 years, the children had higher observed richness than their mothers but lower Shannon and Simpson diversities. At 2 and 5 years, and in the mothers, samples positive for archaea had significantly higher alpha diversity than samples that tested negative for archaea. Mothers with gestational diabetes mellitus (GDM) exhibited a higher relative abundance of *Blautia* but a lower alpha diversity. The variance in offspring gut microbiome explained by maternal GDM became progressively stronger over time, being significant at the age of 5 and explaining 2.5% of the variance.

**IMPORTANCE:**

This study provides the first comprehensive analysis of gut microbiome development in Icelandic children, covering the time from before the introduction of solid foods to 5 years of age. Although the overall developmental patterns of the gut microbiome in Icelandic children were similar to what has been seen in other studies, interesting differences were observed, such as a higher abundance of *Blautia* at an earlier age compared to other study populations. Higher alpha diversity in archaeal-positive samples, both in mothers and in children at the ages of 2 and 5, compared with archaeal-negative samples seen in the present study, is worth further investigation. Additionally, the study suggests a potential role of maternal and perinatal factors, particularly GDM, which was not evident until the age of 5 years, emphasizing the necessity of long-term studies.

## INTRODUCTION

Our bodies are home to trillions of microorganisms, with the colon harboring up to 10^12^ microbial cells per gram of content in adults (1). The bacteria in the colon play a crucial role in maintaining overall well-being, for example, aiding in digestion, protecting against pathogens, and contributing to immune system regulation (2–4). The first 1,000 days, starting at conception, are of special importance in the development of the gut microbiome with implications for health later in life (5, 6). During pregnancy, the placenta is generally considered to be sterile (7), but as soon as the infant is born, multiple external factors in the environment, including the mother’s well-being and gut microbiome, begin to shape the gut microbiome of the infant. Among the factors that impact the development of the gut microbiome are delivery mode (8), antibiotic use (9, 10), type of feeding (human milk vs. formula) (10, 11), the mother’s pre-pregnancy body mass index (BMI) (10), geographical location (12), and transition to solid food (13). During the transition to solid food, the gut microbiome is affected both by the introduction of new foods (13) and the cessation of breast milk or formula (14). During this transition, the gut microbiome becomes more diverse to meet the different challenges of digestion and begins the progression toward an adult-like microbiome, which is often reached at the age of 3–5 years (13, 15).

Diet is one of the strongest influencing factors on the gut microbiome during both childhood and adulthood (13, 16). Different dietary patterns, particularly between Western and non-Western diets, have been studied for their impact on gut microbiome health, with the Western diet being associated with a higher incidence of inflammatory bowel disease (17), metabolic syndrome, obesity, and cardiovascular disease (18, 19). Iceland is an island nation with approximately 397,000 inhabitants, known for its genetically homogeneous population (20). The latest dietary public survey identified that the Icelandic diet is high in fat (36% of total intake), moderate in protein (18%), and low in added sugar (7%) (21).

Although the infant gut microbiome has been extensively studied, research rarely extends beyond the age of 3, leaving later maturation, particularly in underexplored populations with distinct diets, poorly understood. The IceGut cohort addresses this gap by tracking Icelandic mothers and their children from birth to 5 years of age, combining taxonomic and predicted functional profiling to capture changes in diversity, composition, and predicted metabolic potential. Findings show that by age 2, the microbiome approaches an adult-like genus level profile; however, diversity differences persist, underscoring the value of extended follow-up to understand developmental trajectories and their links to maternal and perinatal factors. Furthermore, detailed information on nutritional status and food intake during pregnancy has previously been published for the participating mothers in the present study (22–25), providing valuable information about the study population.

## MATERIALS AND METHODS

### Study population

**Fig 1:**
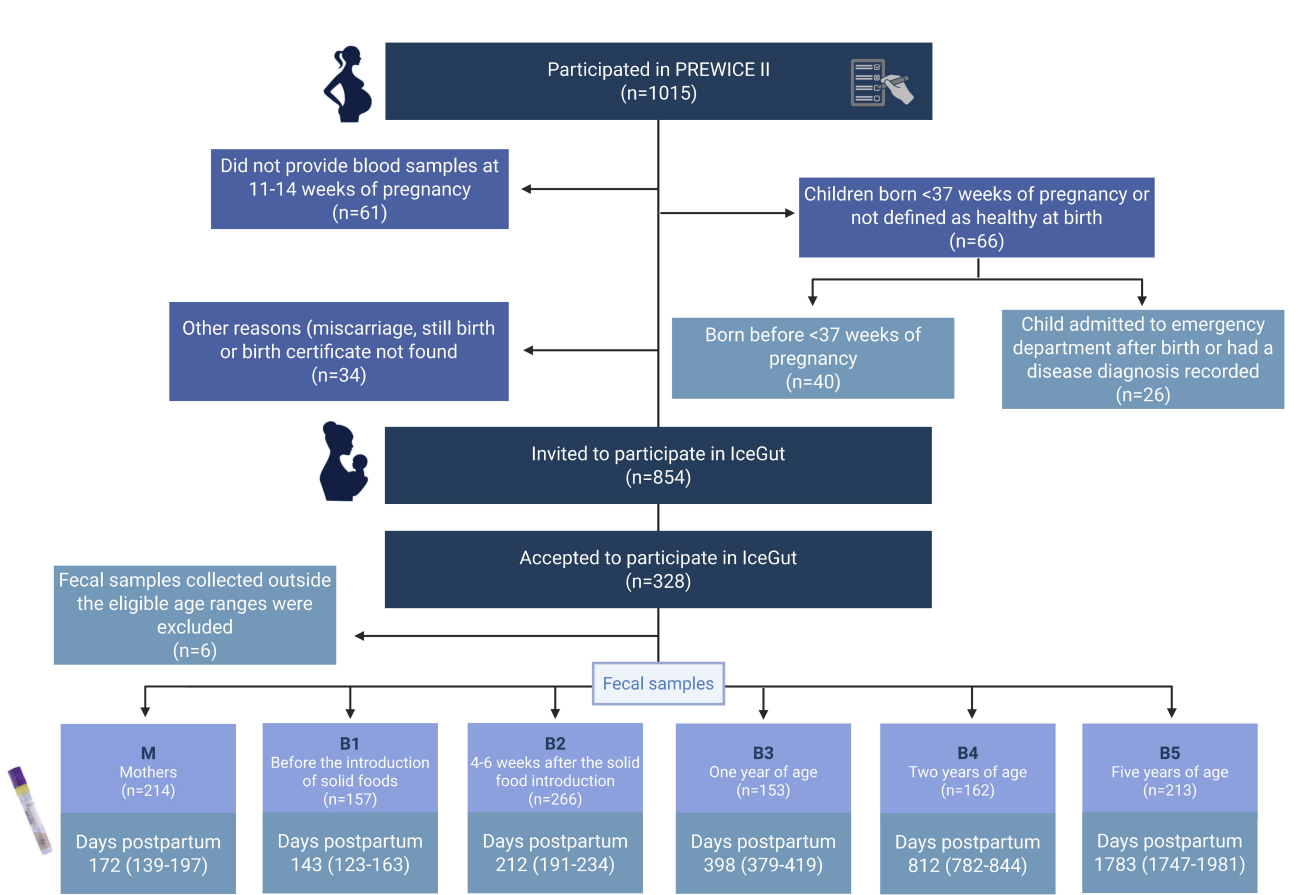
Flow chart of participant recruitment and fecal sample collection of the IceGut cohort. Fecal samples were collected from mothers (M) and children at predefined time points: before solid food introduction (B1), 4–6 weeks after solid food introduction (B2), and at 1 (B3), 2 (B4), and 5 years of age (B5). The ages at sample collection are presented as median days postpartum and interquartile range (Q1–Q3).

The source population comprised women who had participated in the PREWICE II study (Pregnant Women in Iceland II) between October 2017 and March 2018, at the Prenatal Diagnostic Unit at The National University Hospital, Reykjavik, Iceland (22–25). Mothers of full-term healthy infants (≥37 weeks of gestation), who had provided a blood sample in weeks 11–14 of pregnancy, were invited to participate in the follow-up study, the IceGut study (Icelandic Diet and the Infant Gut Microbiome Development). Out of the 1,015 mothers who participated in the PREWICE II study, 854 were invited to participate in the IceGut study, and of these, 328 mother–child pairs accepted (Fig. 1). Women who agreed to participate in the IceGut follow-up study were slightly older than those who did not continue, but there were no differences in their educational level, BMI, or other characteristics at the beginning of pregnancy (26).

**Fig 2:**
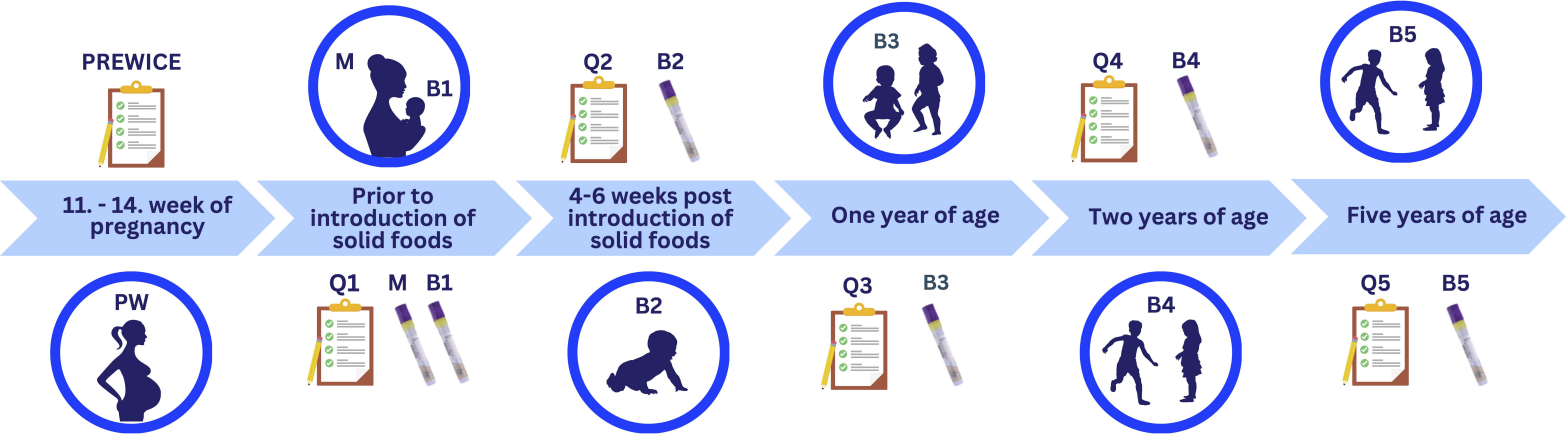
Timeline of sample and data collection. At 11–14 weeks of pregnancy (PW), women completed the PREWICE II questionnaire as part of a larger study. Fecal samples were collected from both mothers (M) and infants (B1) before the introduction of solid food, accompanied by a questionnaire (Q1). Additional fecal samples were collected before the introduction of solid food (B2), at 1 year (B3), 2 years (B4), and 5 years (B5), with corresponding questionnaires (Q2–Q5) at each time point.

Figure 2 outlines the data collection during pregnancy and follow-up. Information about maternal age, marital status, parity, education, height, and pre-pregnancy weight was gathered by a questionnaire during the 11–14 weeks of pregnancy, along with an electronic food frequency questionnaire (FFQ) described by Hrolfsdottir et al. (27). The FFQ assessed the frequency of consumption of 40 food items as well as beverages and dietary supplement intake and has previously been validated against several biomarkers of intake (23, 25, 27, 28). Information on weight gain during pregnancy, complications during pregnancy, and mode of delivery was gathered from hospital records.

### Fecal collection

As shown in Fig. 1 and 2, the participating mothers provided one fecal sample (M) around 6 months post-delivery at the same time as providing either the B1 or B2 sample for their children. About 100 mother–child pairs joined the study at B2, either due to delayed recruitment or because the children had already started solid foods when contacted. The mothers were invited to provide samples from their children at the following time points: before the introduction of solid foods (B1), between 4 and 6 weeks after the introduction of solid foods (B2), at 1 year (B3), at 2 years of age (B4), and at 5 years of age (B5). The number of samples provided at each time point is shown in Fig. 1. The OMNIgene GUT sampling kit (DNA Genotek, Canada) was used for fecal sampling at home by the parents following detailed instructions on the sampling procedure. Upon delivery to the laboratory, and within four weeks of collection, the samples were placed into storage at −80°C.

Information on infant feeding and antibiotic use was collected through questionnaires (Q1–Q5) at all time points (Fig. 2). Because the number of mothers providing samples varied, maternal characteristics (pre- and post-pregnancy) were compared across groups using the Kruskal–Wallis test for continuous or ordinal variables and χ^2^ tests for categorical variables (Table 1). Significance was set at *P* < 0.05.

**TABLE 1:** Maternal characteristics corresponding to each fecal sample time point and the complete series (child samples submitted at all scheduled time points)^*d*^

Parameter	Total cohort (*n* = 328)	B1 (*n* = 157)	B2 (*n* = 266)	B3 (*n* = 153)	B4 (*n* = 162)	B5 (*n* = 213)	M (*n* = 214)	Complete series^*a*^ (*n* = 46)	*P* value
Maternal age (years)									
18–24	47 (14.3)	27 (17.2)	38 (14.3)	22 (14.4)	26 (16.0)	28 (13.1)	27 (12.6)	11 (23.9)	0.595^*b*^
25–29	106 (32.3)	50 (31.8)	85 (32.0)	51 (33.3)	47 (29.0)	69 (32.4)	61 (28.5)	14 (30.4)	
30–34	112 (34.1)	54 (34.4)	92 (34.6)	49 (32.0)	52 (32.1)	71 (33.3)	78 (36.4)	13 (28.3)	
35–39	53 (16.2)	22 (14.0)	42 (15.8)	26 (17.0)	29 (17.9)	37 (17.4)	40 (18.7)	6 (13.0)	
40–45	8 (2.4)	4 (2.5)	8 (3.0)	3 (2.0)	6 (3.7)	6 (2.8)	8 (3.7)	2 (4.3)	
Missing	2 (0.6)	0 (0.0)	1 (0.4)	2 (1.3)	2 (1.2)	2 (0.9)	0 (0.0)	0 (0.0)	
Marital status (*n*)									
Married/cohabitant	314 (95.7)	152 (96.8)	253 (95.1)	145 (94.8)	153 (94.4)	202 (94.8)	206 (96.3)	45 (97.8)	0.932^*c*^
Single	7 (2.1)	3 (1.9)	6 (2.3)	3 (2.0)	5 (3.1)	5 (2.3)	3 (1.4)	0 (0.0)	
Missing	7 (2.1)	2 (1.3)	7 (2.6)	5 (3.3)	4 (2.5)	6 (2.8)	5 (2.3)	1 (2.2)	
Parity (*n*)									
Nulliparous	138 (42.1)	65 (41.4)	109 (41.0)	66 (43.1)	71 (43.8)	89 (41.8)	81 (37.9)	22 (47.8)	0.915^*c*^
Primi/multiparous	189 (57.6)	92 (58.6)	156 (58.6)	86 (56.2)	90 (55.6)	123 (57.7)	133 (62.1)	24 (52.2)	
Missing	1 (0.3)	0 (0.0)	1 (0.4)	1 (0.7)	1 (0.6)	1 (0.5)	0 (0.0)	0 (0.0)	
Mothers’ education (*n*)									
Elementary school or less	27 (8.0)	12 (7.6)	23 (8.7)	9 (5.9)	11 (6.8)	14 (6.6)	15 (7.0)	2 (4.3)	0.989^*b*^
High school and similar	98 (29.9)	52 (33.1)	76 (28.6)	48 (31.4)	53 (32.7)	66 (31.0)	61 (28.5)	16 (34.8)	
Bachelor’s	116 (35.4)	54 (34.4)	92 (34.6)	51 (33.3)	49 (30.2)	80 (37.6)	81 (37.9)	18 (39.1)	
Master’s or doctorate	86 (26.2)	39 (24.8)	74 (27.8)	44 (28.8)	48 (29.6)	52 (24.4)	57 (26.6)	10 (21.7)	
Missing	1 (0.3)	0 (0.0)	1 (0.4)	1 (0.7)	1 (0.6)	1 (0.5)	0 (0.0)	0 (0.0)	
Mothers’ height (m)									
Mean [SD]	1.68 [0.06]	1.68 [0.06]	1.67 [0.06]	1.68 [0.06]	1.68 [0.06]	1.67 [0.05]	1.68 [0.05]	1.68 [0.05]	0.999^*b*^
Median [Q1, Q3]	1.67 [1.64–1.71]	1.67 [1.64–1.71]	1.67 [1.64–1.71]	1.67 [1.64–1.71]	1.67 [1.64–1.71]	1.67 [1.64–1.70]	1.67 [1.64–1.71]	1.67 [1.65–1.70]	
Missing	1	0	1	1	1	1	0	0	
Mothers’ pre-pregnancy weight (kg)									
Mean [SD]	71.32 [15.92]	69.92 [13.99]	72.04 [16.47]	71.44 [17.59]	72.30 [17.51]	72.11 [16.32]	72.65 [17.09]	71.37 [15.22]	0.893^*b*^
Median [Q1, Q3]	68.00 [60.00–78.00]	67.00 [60.00–77.00]	68.00 [60.00–80.00]	67.00 [60.00–78.00]	68.00 [60.00–80.00]	69.00 [60.75–79.00]	69.00 [60.00–80.00]	68.50 [60.00–77.00]	
Missing	3	2	1	1	1	1	2	0	
Total weight gain during pregnancy									
Mean [SD]	12.73 [5.44]	13.13 [4.91]	12.37 [5.56]	12.02 [4.52]	12.38 [5.24]	12.70 [5.57]	12.83 [5.31]	12.15 [4.52]	0.681^*b*^
Median [Q1, Q3]	12.40 [9.00–15.90]	13.55 [9.30–16.00]	12.00 [8.90–15.40]	11.80 [9.03–15.18]	12.20 [9.00–15.50]	12.30 [9.15–15.75]	12.90 [9.20–16.00]	12.40 [9.15–15.33]	
Missing	39	15	31	23	25	30	29	6	
Mothers’ pre-pregnancy BMI									
<18.5	3 (0.9)	2 (1.3)	2 (0.8)	2 (1.3)	0 (0.0)	0 (0.0)	1 (0.5)	0 (0.0)	0.885^*b*^
18.5–24.99	177 (54.0)	90 (57.3)	138 (51.9)	82 (53.6)	87 (53.7)	111 (52.1)	109 (50.9)	25 (54.3)	
25–29.99	91 (27.7)	38 (24.2)	76 (28.6)	41 (26.8)	47 (29.0)	64 (30.0)	61 (28.5)	12 (26.1)	
≥30	54 (16.5)	25 (15.9)	49 (18.4)	27 (17.6)	27 (16.7)	37 (17.4)	41 (19.2)	9 (19.6)	
Missing	3 (0.9)	2 (1.3)	1 (0.4)	1 (0.7)	1 (0.6)	1 (0.5)	2 (0.9)	0 (0.0)	
Cesarean									
No	288 (87.8)	142 (90.4)	232 (87.2)	134 (87.6)	143 (88.3)	185 (86.9)	186 (86.9)	42 (91.3)	0.959^*c*^
Yes	40 (12.2)	15 (9.6)	34 (12.8)	19 (12.4)	19 (11.7)	28 (13.1)	28 (13.1)	4 (8.7)	
Missing	0	0	0	0	0	0	0	0	
Gestational diabetes mellitus									
No	238 (72.6)	112 (71.3)	194 (72.9)	105 (68.6)	108 (66.7)	151 (70.9)	152 (71.0)	30 (65.2)	0.987^*c*^
Yes	40 (12.2)	19 (12.1)	33 (12.4)	16 (10.5)	22 (13.6)	27 (12.7)	25 (11.7)	7 (15.2)	
Missing	50 (15.2)	26 (16.6)	39 (14.7)	32 (20.9)	32 (19.8)	35 (16.4)	37 (17.3)	9 (19.6)	

^*a*^ The mother and child pairs that had all five fecal samples from the children (B1, B2, B3, B4, and B5).

^*b*^ Test for continuous and ordinal variables and χ^2^ test.

^*c*^ For categorical variables.

^*d*^ *P* value is calculated by Kruskal-Wallis test.

### DNA extraction, polymerase chain reaction (PCR) amplification and sequencing

DNA was isolated from 250 mg of thawed fecal samples using the QIAamp PowerFecal Pro DNA Kit (QIAGEN, Germany) according to the manufacturer’s instructions. The DNA concentration and purity were determined using a NanoDrop ND-1000 Spectrophotometer (Thermo Fisher Scientific, USA), and the DNA was stored at −20°C until the stage of 16S rRNA gene amplification and library preparation. Partial 16S rRNA gene amplification was performed using primers 515F (29) and 806R (30), which are primarily designed to target the bacterial V4 region but also amplify a subset of archaeal taxa. PCR was carried out in a 25 µL reaction volume containing 10 ng of DNA, 0.5 U of high-fidelity Q5 DNA polymerase (New England Biolabs, USA), 1× Q5 reaction buffer, 200 µM dNTPs (New England Biolabs, USA), and 0.5 µM of each primer containing Illumina (Illumina, USA) overhang adapters. Amplification was performed on a MiniAmp Thermal Cycler (Thermo Fisher Scientific, USA) with an initial denaturation at 98°C for 30 s, followed by 30 cycles of 98°C for 10 s, 52°C for 30 s, and 72°C for 30 s, with a final extension at 72°C for 2 min. To monitor the accuracy of amplification and sequencing, a subset of fecal samples was re-sequenced across multiple sequencing runs, and positive (ZymoMock_6331, Zymo Research, USA) and negative PCR controls (sterile deionized water) were included in the workflow. Amplicon size was verified by agarose gel electrophoresis using 1% agarose gels in 1× TAE buffer, stained with SYBR Safe DNA gel stain (Invitrogen, USA). Each sample was amplified in triplicate, and the three reactions were pooled before PCR purification using the HighPrep PCR Clean Up System (MagBio Genomics, USA), following the manufacturer’s instructions. Library preparation was performed as described by Leeper et al. (31), and sequencing was conducted on a MiSeq System (Illumina, USA) using V3 chemistry with 2 × 300 cycles.

### Microbial community analysis

Microbial community analyses were performed in R version 4.1.2 (32) implemented in Rstudio version 4.1.2 (RStudio, version 4.4.1) and Bioconda packages in Python (33). Raw FASTQ files were pre-processed to produce amplicon sequence variants (ASVs) using the DADA2 package version 1.14.2 (34). Variables in the function filterAndTrim were set to truncLen = c(250,200), trimLeft = c(30,30), maxN = 0, maxEE = c(2,2), truncQ = 2. Taxonomic classification of the ASVs was performed against the SILVA database version 138.2 (35).

Manual decontamination of the ASV table was performed by removing 18 contaminant sequences abundant in negative controls but rare in samples. ASVs from eukaryotic, chloroplast, and mitochondrial origins were also filtered out. The samples were normalized through rarefaction to a depth of 16,071 sequences per sample, using the rarefy_even_depth() function from the phyloseq package (version 1.48.0) (36) in R.

Taxonomic composition bar plots were created with the phyloseq package. Taxa were aggregated at the genus and phylum levels into relative abundances using rarefied data. “Not Available” (NA) genera were reassigned to their corresponding family. In cases where “NA” persisted at the family level, taxa were further classified at the order level, followed by class, phylum, and, if necessary, kingdom. Taxa with <1% total relative abundance in at least one of the groups were grouped as the “other” category.

Alpha diversity was assessed in rarefied data using the observed species, Shannon, and Simpson indices, using the estimate_richness() function from the phyloseq package (version 1.48.0) in R. The stat_compare_means() function with the method set to wilcox.test from the ggpubr package (version 0.6.0) (37) was used to perform the Wilcoxon test for comparing groups. However, for comparisons of observed archaeal groups with respect to B5, a Student’s *t*-test was applied.

Beta diversity was evaluated in rarefied data using principal coordinates analysis (PCoA) with the Jaccard dissimilarity distance measure, using plot_ordination() function from the phyloseq package in R. A PERMANOVA test was conducted with the number of permutations = 999 and Jaccard as a method, and using the adonis2() function from the vegan package (version 2.6.10) (38) in R.

Differential abundance analysis between the groups of age (B1, B2, B3, B4, B5, and M) was conducted using the ancombc2() function in R from the Analysis of Composition of Microbiomes with Bias Correction (ANCOMBC) package (version 2.6.0) (39, 40) and pairwise = TRUE on unrarefied data. The analysis was performed at the genus level and adjusted using the Holm-Bonferroni method (41).

Microbial functional pathways and Kyoto Encyclopedia of Genes and Genomes (KEGG) orthologs were predicted using Phylogenic Investigation of Communities by Reconstruction of Unobserved States 2 (PICRUSt2) (42). Analysis was conducted using the picrust2 bioconda package version 2.6.1 by running the picrust2_pipeline.py script.

Taxonomic and predicted functional pathway trajectory analysis across age groups was performed using the TcGSA.LR() and plot1GSA() functions from the Time-course Gene Set Analysis (TcGSA) package (v 0.12.10) (43) in R. These functions fit linear mixed models to time-series data, accounting for interindividual variability and cluster features based on shared temporal patterns. Taxonomic analysis included ASVs identified as significantly different across age groups by ANCOMBC2. Representative ASVs were visualized by mean log_10_ relative abundance using the ggplot2 (v 3.5.1) (44) in R. Predicted functional pathway trajectory analysis was conducted using the pred_metagenome_unstrat output table from the PICRUSt2 analysis, which contained predicted KEGG pathway abundances for each sample. Temporal trends were assessed across age groups using TcGSA, and the individual KEGG orthologs contributing to each pathway were evaluated to characterize pathway-level dynamics over time.

To assess the relationship between measured variables and community composition, the envfit() function from the vegan package (v 2.6.10) in R was used. The method fits environmental vectors on a non-metric multidimensional scaling (NMDS) ordination space. The statistical significance of each variable was evaluated using permutation tests (*n* = 999), with *R*^2^ values used to assess the strength of the association.

## RESULTS

### Cohort characteristics and descriptive data of the study population

Fecal samples were collected from 214 mothers, and samples were provided from 328 children at different time points, resulting in 1,165 samples in total. Complete sets of maternal and child samples across all time points (M, B1–B5) were available for 43 mother–child pairs, and 46 children contributed samples at all child time points (B1–B5). The mean maternal age at delivery was 30–34 years, the mean pre-pregnancy BMI was 25 kg/m², and the mean gestational weight gain was 12.7 kg. Gestational diabetes mellitus (GDM) was diagnosed in 12.2% of mothers, and 12.2% of the children were delivered via cesarean section. The characteristics of mothers providing samples at different time points are shown in Table 1, with no difference observed between groups providing samples for their children at different time points. The maternal frequency of intake of food and beverages in early pregnancy was also similar between groups (Table S1). Therefore, assuming that variation in infant gut microbiota over time is not attributable to differences in maternal characteristics or diet during pregnancy, information on antibiotic intake within each group is provided in Table S2. Among those who provided the fecal sample before the introduction of solid food (B1), 99.3% were receiving some level of breastfeeding, whereas by 5 years (B5), none were still being breastfed (Table S3).

### Maternal gut microbiome diversity around 6 months postpartum

**Fig 3:**
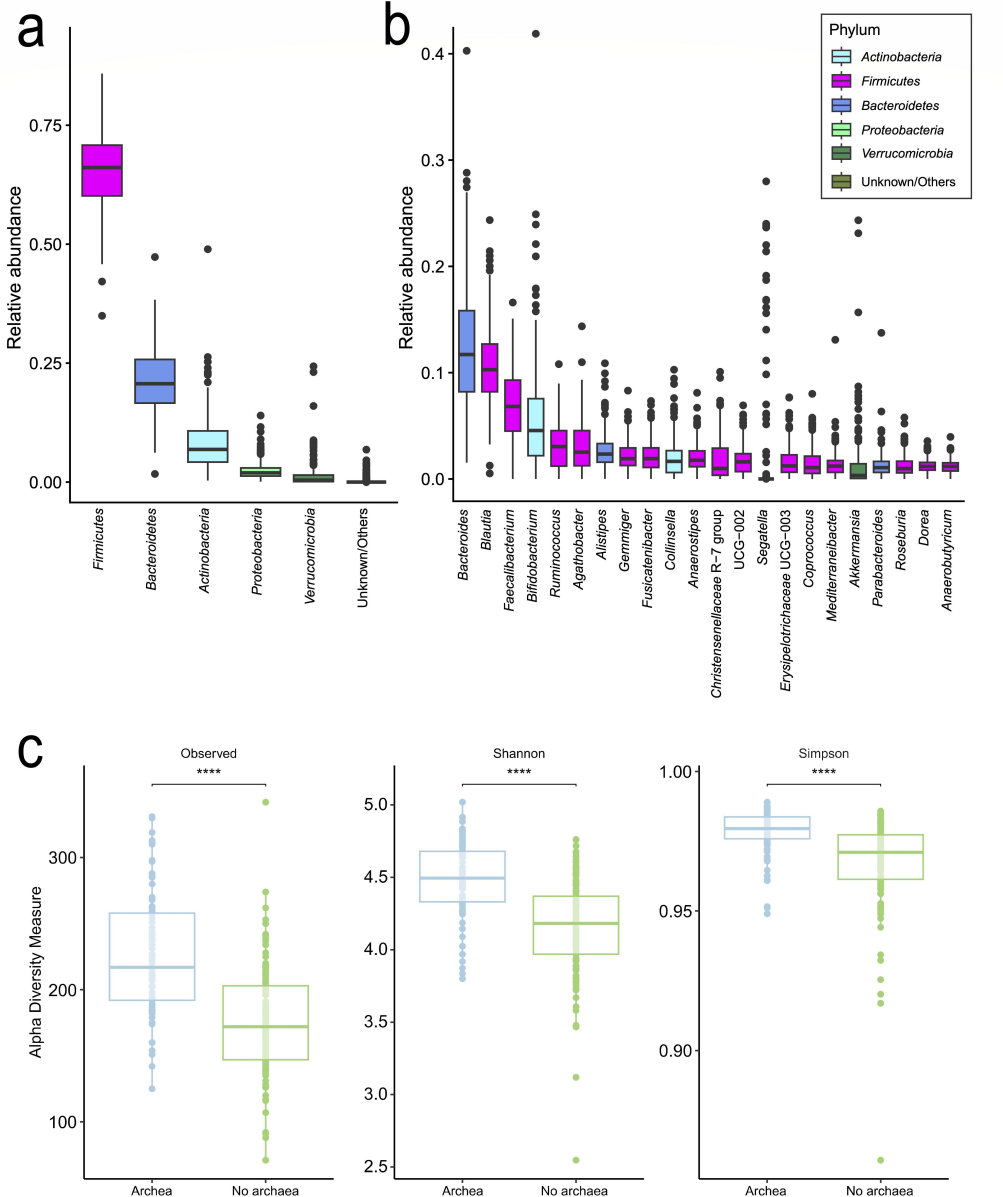
Relative abundances of bacterial phyla (**a**) and genera (**b**) in all maternal fecal samples. Genera are color-coded according to their corresponding phyla. (**c**) Comparison of alpha diversity in maternal samples with and without detected archaea.

The maternal gut microbiome was dominated by four phyla, accounting for 97.6% of the total relative abundance: *Firmicutes* (also recognized as *Bacillota*) (65.6%), *Bacteroidetes* (also recognized as *Bacteroidota*) (21.3%), *Actinobacteria* (also recognized as *Actinomycetota*) (8.3%), and *Proteobacteria* (also recognized as *Pseudomonadota*) (2.4%) (Fig. 3a). Less abundant phyla included *Verrucomicrobia* (also recognized as *Verrucomicrobiota*) (1.4%) and *Thermodesulfobacteria* (also recognized as *Thermodesulfobacteriota*) (0.5%). At the genus level, the microbiome was dominated by four genera (35.8%), with *Bacteroides* being the most abundant (12.7%), followed by *Blautia* (10.7%), *Faecalibacterium* (6.8%), and *Bifidobacterium* (5.7%) (Fig. 3b). Archaea (*Methanosphaera* (0.01%), *Methanobrevibacter* (0.22%), *Methanomassiliicoccus* (<0.01%), and *Candidatus Methanogranum* (<0.01%) were detected in 87 out of the 214 maternal samples (40.7%). Maternal samples positive for archaea had a significantly (*P* < 0.001) higher Shannon diversity, Simpson diversity, and observed ASVs compared with maternal samples without detected archaea (Fig. 3c).

### Development of infant gut microbiome diversity up to 5 years of age

**Fig 4:**
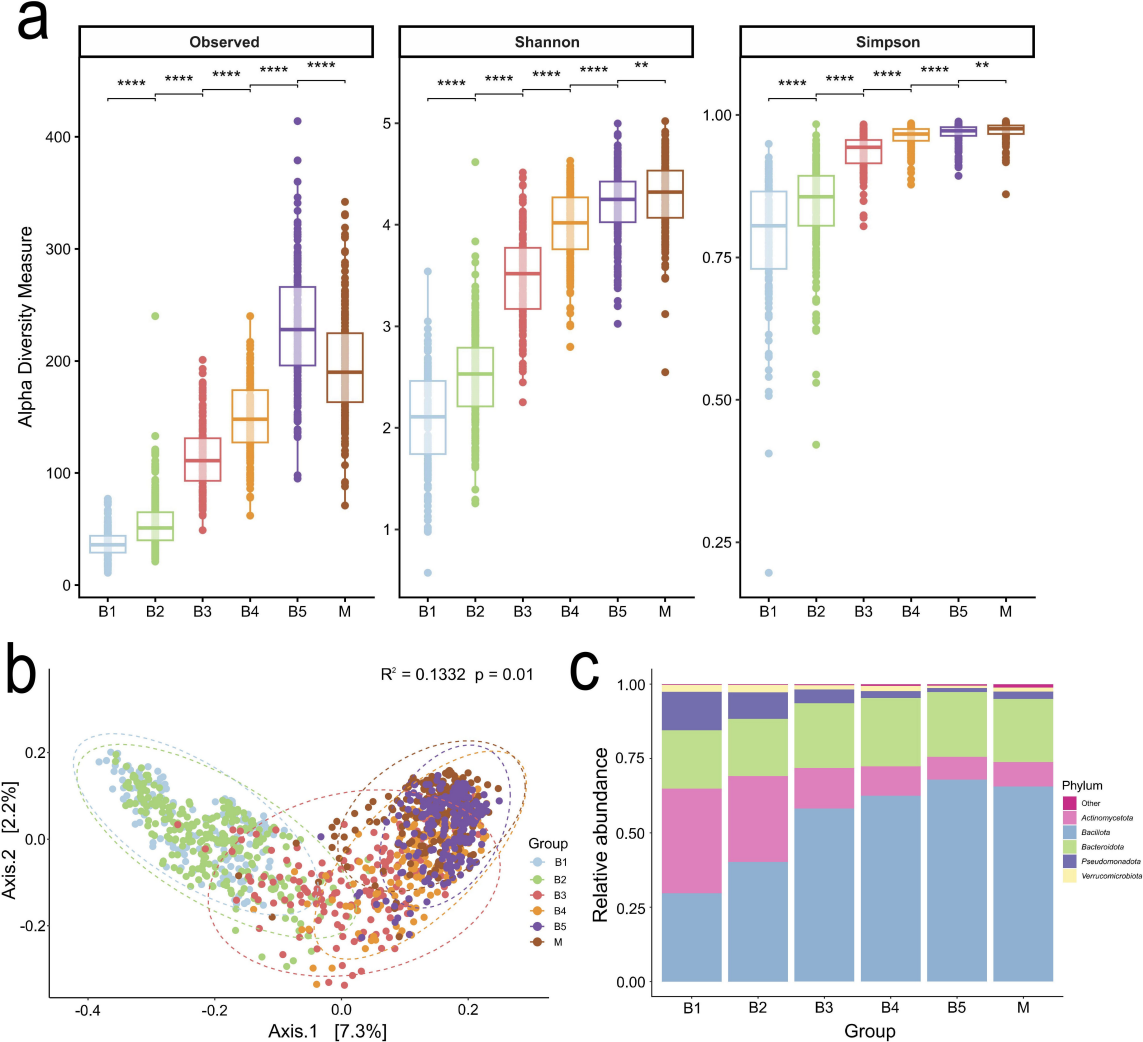
Development of the infant gut microbiome until 5 years of age based on rarefied data. (**a**) Alpha diversity (observed, Shannon, and Simpson indices) of gut microbiome for each age group (B1, B2, B3, B4, B5, and M). Statistical significance was assessed by the Wilcoxon test, and *P* < 0.05 was considered statistically significant, as shown by asterisks (**, <0.01; ***, <0.001; ****, <0.0001). (**b**) PCoA plot at the genus level, based on Jaccard dissimilarities, illustrating the beta diversity among the groups. The statistical difference was determined using pairwise Adonis. (**c**) Bar plots presenting the relative abundances of the five most prevalent phyla within each age group, with phyla below 1% grouped as "other."

All alpha diversity metrics for the infant gut microbiome increased significantly across age groups. Observed ASVs were even richer in B5 than the mothers’ group (Fig. 4a). Beta diversity analysis showed that the B1 and B2 samples clustered together, whereas the B4, B5, and M samples formed a distinct cluster, with B3 positioned between these two sub-groups (Fig. 3b). The most abundant phyla were *Firmicutes*, *Bacteroidetes, Actinobacteria*, and *Proteobacteria* in all age groups. *Firmicutes* and *Bacteroidetes* increased with age, whereas *Actinobacteria* and *Proteobacteria* decreased with age (Fig. 4c).

**Fig 5:**
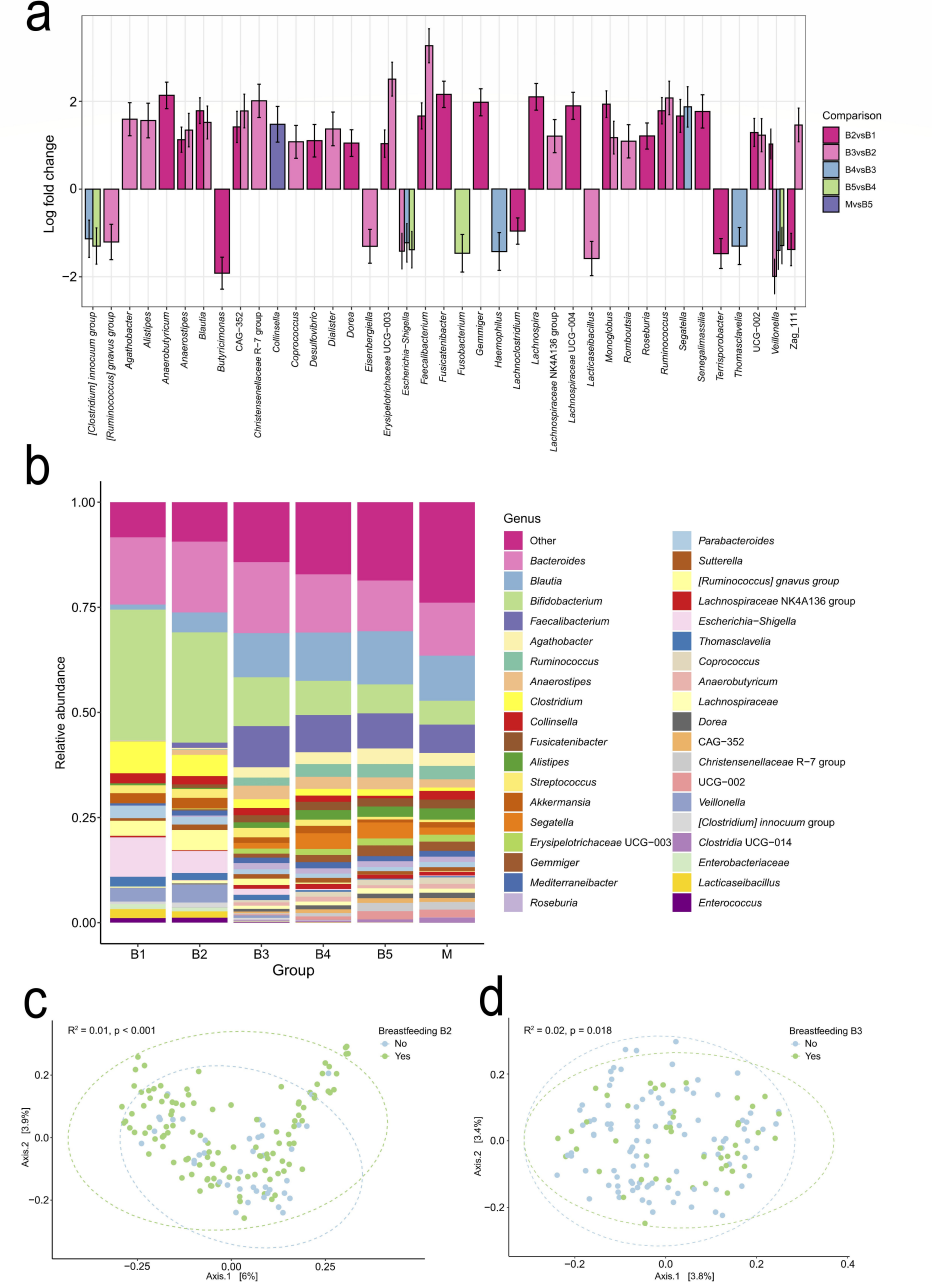
(**a**) Differential abundance analysis (ANCOM-BC2) of all genera that were differentially abundant between the compared groups based on unrarefied data. (**b**) Relative abundance of all genera of the maternal and infant gut microbiome until 5 years of age based on rarefied data. Only genera detected at a relative abundance greater than 1% in at least one group are shown. Genera with relative abundances below 1% in all groups are combined into the category "other." (**c**) PCoA plot at the genus level, based on Jaccard dissimilarities based on rarefied data, illustrating the beta diversity between infants who were still being breastfed 4–6 weeks after the introduction of solid foods (B2) and those who were not. (**d**) PCoA plot at the genus level, based on Jaccard dissimilarities, illustrating the beta diversity between infants who were being breastfed at 1 year of age (B3) and those who were not (B3).

Differential abundance analysis showed notable shifts in genera across most age groups (Fig. 5a). Here, below, genera with relative abundances exceeding 1% are highlighted. Between B1 and B2, *Bifidobacterium* remained the dominant genus in both groups, followed by *Bacteroides* and *Escherichia-Shigella* (Fig. 5b), whereas the genera *Blautia*, *Faecalibacterium*, *Veillonella*, and *Anaerostipes* increased significantly. From B2 to B3, further increases were observed in *Blautia*, *Faecalibacterium*, and *Anaerostipes*, as well as increases in *Alistipes*, *Agathobacter*, *Ruminococcus*, and *Erysipelotrichaceae* UCG-003, whereas *Escherichia-Shigella*, *Veillonella*, *Lacticaseibacillus*, and the [*Ruminococcus*] *gnavus* group decreased. Notably, significant differences in overall community composition between breastfed and non-breastfed children were observed for B2 (PERMANOVA, Jaccard distance: *R*² = 0.01, *P* < 0.001) and B3 (*R*² = 0.02, *P* = 0.018) (Fig. 5c and d). Between 1 and 2 years of age (B3 and B4), differential abundances of *Segetella* increased, whereas *Escherichia-Shigella* and *Thomasclavelia* declined. By B4 and B5, the microbial composition more closely resembled that of the mothers, with *Bacteroides* emerging as the dominant genus, followed by *Blautia*, *Faecalibacterium*, and *Bifidobacterium*. No significant differences were observed in the differential abundance between B4 and B5. Comparison between B5 and M showed higher relative abundance in *Collinsella*, whereas no significant changes were found between B4 and M (Fig. 5a). Full lists of genera and phyla with relative abundances above 1% are provided in Tables S4 and S5, respectively.

### Infant gut microbiome and predictive functional signatures up to 5 years of age

**Fig 6:**
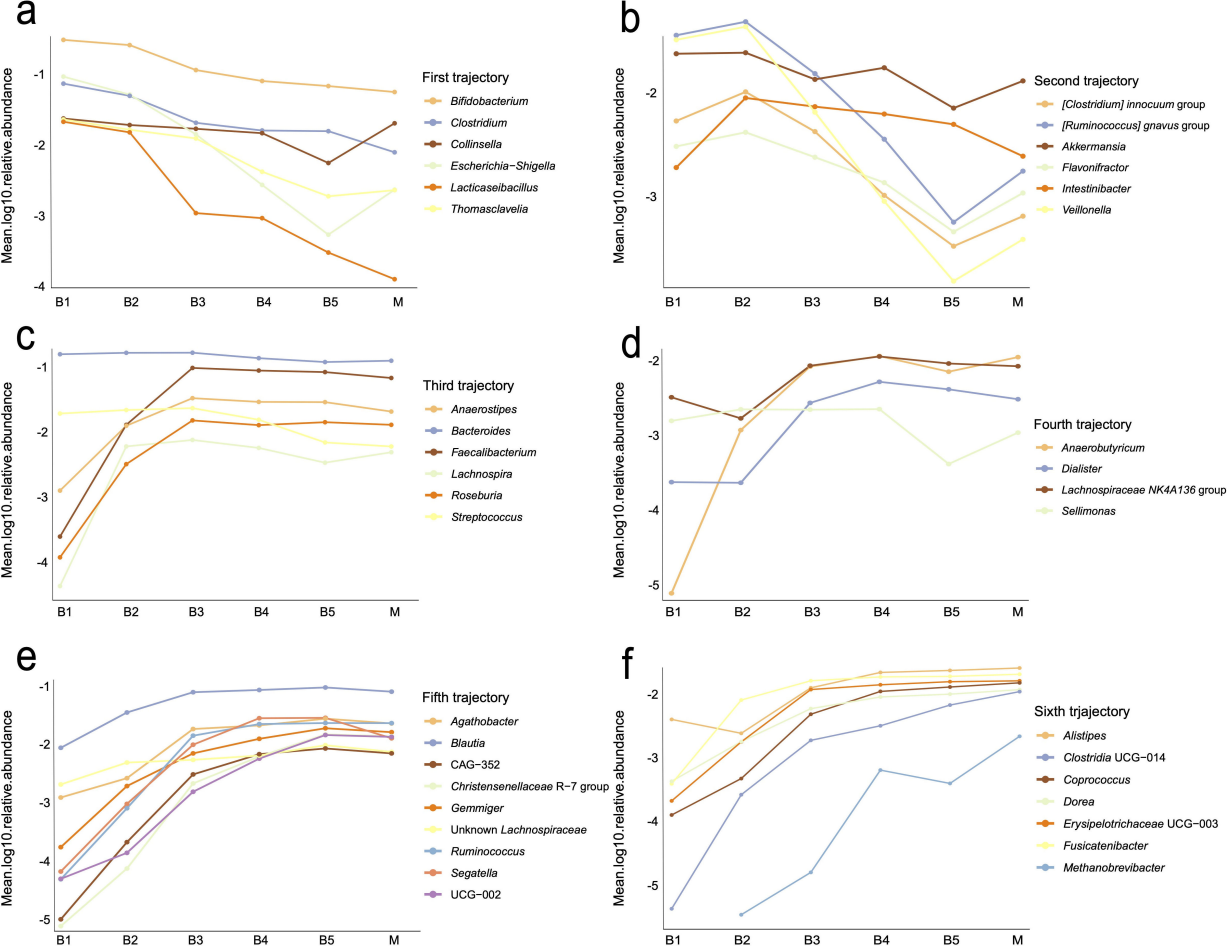
Trajectories of microbiome development of the infant gut microbiome until 5 years of age, including maternal samples (**a–f**). Individual trajectories of genera exhibiting statistically significant differences between any two timepoints as determined by ANCOM-BC2. Each trajectory highlights the genera showing peak relative abundance at specific ages. (**a**) Trajectory 1, before the introduction of solid foods (B1). (**b**) Trajectory 2, 4–6 weeks after solid food introduction (B2). (**c**) Trajectory 3, at one year of age (B3). (**d**) Trajectory 4, at 2 years of age (B4). (**e**) Trajectory 5, at 5 years of age (B5). (**f**) Trajectory 6, mothers (M).

**Fig 7:**
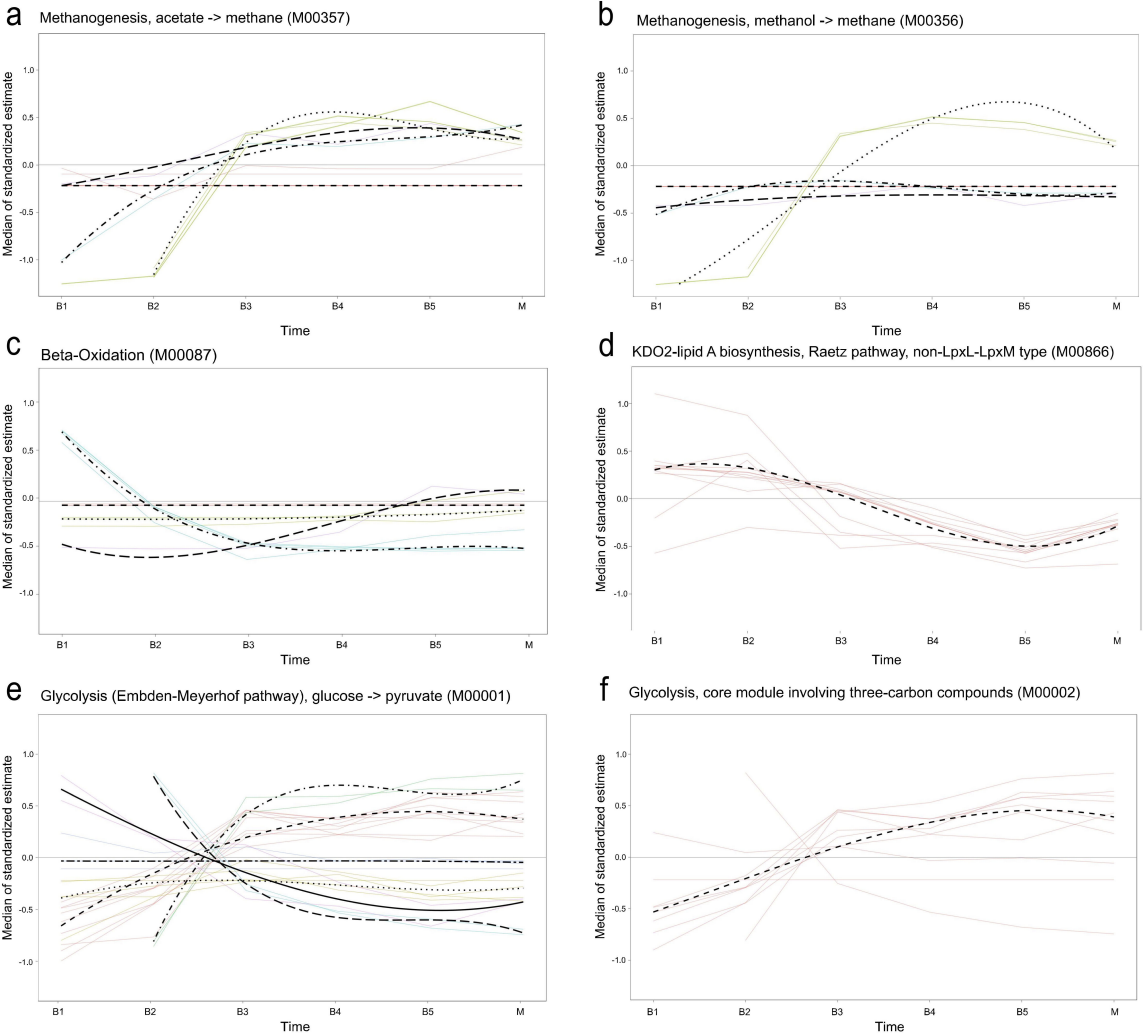
Temporal trajectories of standardized predicted gene count patterns for selected microbial functional modules across developmental stages of the infant gut microbiome until 5 years of age, including maternal samples. Trajectories were inferred using time-course gene set analysis (TcGSA). Non-dotted colored lines represent individual KEGG orthologs (KEGGs) within each metabolic pathway. Colors indicate distinct trajectory clusters based on KEGG expression dynamics. Dotted lines represent the average expression trend of each cluster over time. (**a**) Methanogenesis (acetate → methane). (**b**) Methanogenesis (methanol → methane). (**c**) Beta-oxidation. (**d**) KDO2-lipid A, Raetz pathway, non-LpxL-LpxM type. (**e**) Glycolysis (Embden-Meyerhof pathway, glucose → pyruvate) and (**f**) glycolysis, core module involving three-carbon compounds.

All samples, including maternal samples, were used to further characterize the developmental trajectories of the gut microbiome from stages B1 through B5, resulting in six distinct trajectories (Fig. 6a through f). The first trajectory included genera with a peak relative abundance before the introduction of solid foods (B1), such as *Bifidobacterium*, *Escherichia-Shigella*, *Clostridium*, *Collinsella*, *Thomasclavelia*, and *Lacticaseibacillus* (Fig. 6a). The second trajectory included genera that peaked 4–6 weeks after the introduction of solid food (B2), including [*Ruminococcus*] *gnavus group*, *Veillonella*, *Akkermansia*, [*Clostridium*] *innocuum* group, *Intestinibacter*, and *Flavonifractor* (Fig. 6b). At 1 year (B3), *Bacteroides*, *Faecalibacterium*, *Anaerostipes*, *Streptococcus*, *Roseburia*, and *Lachnospira* reached peak abundance (Fig. 6c). This transition was associated with notable changes in predicted functional gene counts related to methane production, beta-oxidation, the Raetz pathway (non-LpxL-LpxM type), and glycolysis (Fig. 7a through f). Specifically, all predicted gene clusters involved in methane production increased (Fig. 7a and b), whereas the Raetz pathway showed a decrease across all clusters (Fig. 7d). In contrast, beta-oxidation and glycolysis exhibited mixed patterns, with some gene clusters decreasing and others increasing (Fig. 7c, e, and f). At 2 years of age (B4), *Sellimonas*, *Dialister*, *Anaerobutyricum*, and unknown *Lachnospiraceae* NK4A136 group reached peak abundance (Fig. 6d). By 5 years of age (B5), when the observed alpha diversity was at its highest, *Blautia*, *Segatella*, *Agathobacter*, *Ruminococcus*, *Gemmiger*, *Christensenellaceae* R-7 group, and UCG-002 peaked in relative abundance (Fig. 6e). No notable changes were detected in key predicted metabolic pathways for B4, B5, and M. The mothers had the highest Shannon and Simpson diversity, indicating a more even distribution of bacterial taxa, with *Alistipes*, *Fusicatenibacter*, *Erysipelotrichaceae* UCG-003, *Coprococcus*, *Dorea*, *Clostridia* UCG-014, and *Methanobrevibacter* peaking in relative abundance (Fig. 6f). A list of all significantly different pathways between age groups can be found in Table S6.

### Pre- and postnatal factors associated with infant gut microbiome development until 5 years of age

**Fig 8:**
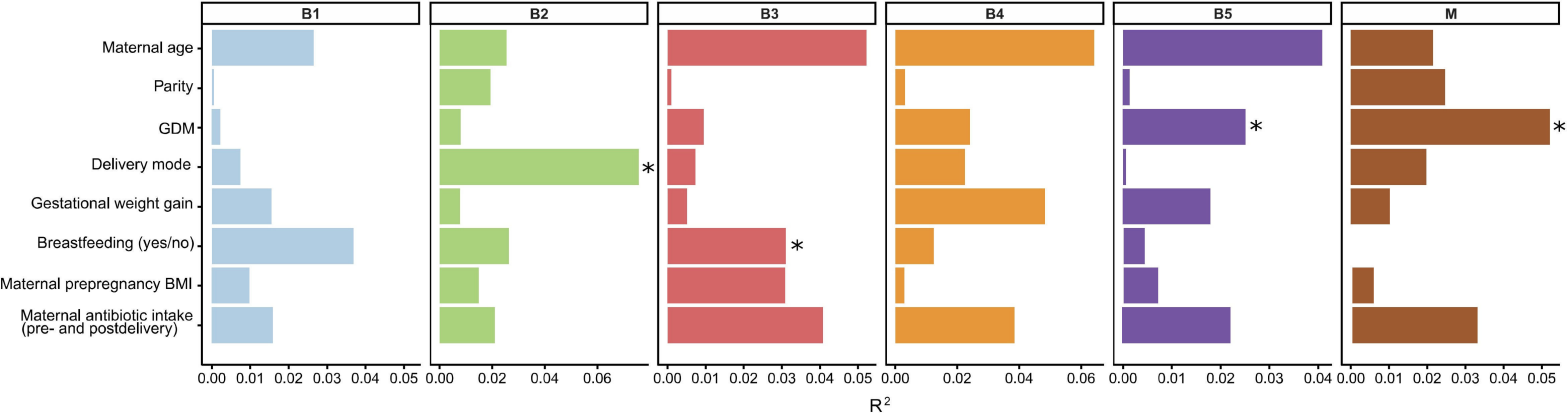
Associations of host factors on microbial community variation of the infant gut microbiome until 5 years of age, including maternal samples. Significant differences (*P* < 0.05) are noted with *. R² represents the percentage of variation in microbiome composition that is explained by each variable.

Pre- and postnatal factors were fitted onto the ordination space using envfit to assess their relation to microbial community variation (Fig. 8). The delivery mode explained about 8% of the variance in sample distribution at B2 (*R*² = 0.076, *P* = 0.001), and breastfeeding accounted for about 3% of the variance at the age of 1 year (B3) (*R*² = 0.031, *P* = 0.044). The variance in offspring gut microbiome explained by maternal GDM became progressively stronger over time and significant at the age of 5 (B5) (*R*² = 0.025, *P* = 0.026), but no significant associations were found for GDM at earlier time points. However, maternal GDM explained approximately 5% of the variance in the mothers' gut microbiome (M) (*R*² = 0.052, *P* = 0.010). Within the IceGut cohort, mothers with diagnosed GDM had a higher relative abundance of *Blautia* (*P* = 0.018) compared with those without GDM, as determined by the Wilcoxon rank-sum test. Also, observed ASVs, Shannon and Simpson diversity indices among GDM mothers were significantly lower (*P* = 0.0003, 0.003, and 0.04, respectively) than for those who did not have GDM (Fig. S1).

## DISCUSSION

The current IceGut study provides a comprehensive analysis of the gut microbiome development in Icelandic children from before the introduction of solid food to 5 years of age, as well as an analysis of the maternal gut microbiome at around 6 months postpartum. This work presents the first gut microbiome cohort of Icelandic children, thereby addressing a geographic gap, as Iceland remains underrepresented in microbiome studies. By extending sampling beyond the commonly studied first 3 years of life, and including both taxonomic and predicted functional profiling, the present study offers valuable insights into the later stages of microbiome maturation and their potential associations with maternal characteristics and perinatal factors.

The maternal gut microbial composition was mostly consistent with the findings from other studies on 16S rRNA data, as discussed in a systematic review of the postpartum period (45). Notably, *Actinobacteria* had a relatively high abundance in the IceGut study (8.3%) compared to <3% in Colombians (46), South Koreans (47, 48), Americans (46), Chileans (49), Germans (48), and Spaniards (50), but higher values were reported from Japanese (46) populations. High abundance of *Actinobacteria* may be affected by dietary habits in Iceland, where cod liver oil is commonly consumed, providing vitamin A, a nutrient previously linked to higher *Actinobacteria* abundance (21, 51) or pregnancy-related shifts (45), although the contribution of either or both remains uncertain. At the genus level, *Blautia* was prevalent in all maternal samples, with an average relative abundance of 10.7%, much higher than Italy (52), Vietnam (53), Sweden (54), or the USA (55) but lower than Japan (56). Notably, fecal sample collection, sample storage, DNA extraction, primers, or PCR protocols can vary across studies and can affect detected taxa and relative abundances. Therefore, methodological differences cannot be ruled out as a contributing explanation for these between-study differences.

In contrast with prior studies, including a recent Swedish cohort that examined similar age groups and used the same primer pair as in the present study (57), the children in the IceGut study exhibited a higher observed richness (ASVs) at the age of 5 than their mothers. However, the Shannon and Simpson diversity indices were significantly higher in the maternal samples (Fig. 4a), suggesting that although the gut microbiome of 5-year-old children may contain more taxa, the maternal microbiome is more even in distribution. One possible explanation is the timing of maternal sampling. In the IceGut cohort, the maternal microbiome may still have been recovering from pregnancy-related shifts, which are known to reduce diversity and increase interindividual variability (58). These changes often include a decrease in *Faecalibacterium* and an increase in *Actinobacteria* and *Proteobacteria* (45). Recovery toward a pre-pregnancy state typically occurs within 6–12 months postpartum but is highly individualized and may remain incomplete (45, 58, 59). As pre-pregnancy samples were not available, it cannot be confirmed whether this pattern was present in the IceGut cohort.

In accordance with the results of other studies, the infant gut microbiome in the IceGut cohort showed clear age-related shifts in diversity and composition, with a gradual transition from a *Bifidobacterium*-dominated early profile toward a more adult-like configuration by 2 years of age (Fig. 5b). Alpha diversity increased steadily across time points (Fig. 4a), and beta diversity patterns reflected discrete clustering before and after the introduction of solid foods, with 1 year of age marking a key transitional stage (Fig. 4b). By the ages of 2 years (B4) and 5 years (B5), the microbial community structure closely resembled that of mothers, although some differences persisted in diversity metrics and specific taxa. Interestingly, the infant gut microbiome in the present study displayed a notably high relative abundance of *Blautia* compared with cohorts from Sweden, China, and Ireland, particularly from B2 (4–6 weeks after solid food introduction) and onward (60–64). By 1 year of age (B3), *Blautia* abundance had increased to over 10%, similar to the mothers, with levels remaining stable at 2 years of age (B4) and 5 years (B5). This *Blautia-*dominant pattern resembles the *Blautia* enterotype described in older children and adults, which has been associated with beneficial metabolic traits and higher bacterial diversity but has also been linked to Th22 cell activity and atopic dermatitis (65).

Beta diversity (Fig. 4b), the relative abundance data (Fig. 5a and b), and trajectory analyses (Fig. 6a through f) collectively indicate that 1 year of age marks a critical period in the gut microbiome maturation of this cohort. This is consistent with previous studies demonstrating the progressive colonization and diversification of the gut microbiome during early childhood (3, 5, 8, 66). Functional predictions revealed metabolic shifts during this period, including changes in methane production, β-oxidation, Raetz pathway non-LpxL-LpxM type, and glycolysis (Fig. 7a through f). These changes probably reflect adaptations to dietary diversification, cessation of breastfeeding, and increasing gut microbiome complexity (Fig. 5c and d). A notable observation was the decline in predicted functional gene counts related to β-oxidation, a key lipid metabolism pathway, suggesting reduced bacterial involvement in fatty acid breakdown. This shift could be linked to changes in dietary composition, such as a higher carbohydrate intake relative to fats (67), cessation of breast milk, or the maturation of host-driven lipid metabolism. Conversely, changes in glycolysis pathways indicate that the gut microbiome is adapting to altered carbohydrate utilization. The decline in predicted functional gene count in the Raetz pathway, non-LpxL-LpxM type, may reflect a developmental window in which the immune system is trained in the presence of low-inflammatory stimuli, potentially supporting immune tolerance without triggering harmful inflammation (68). These predictions inferred from 16S rRNA profiles with PICRUSt2 offer putative functional insights, whereas direct metagenomic sequencing provides greater accuracy and resolution.

Emerging evidence suggests that GDM may be linked to long-term variation of offspring microbiome composition through vertical transmission or metabolic programming (69, 70). In the present study, the presence of maternal GDM was found to explain about 2.5% of the variance in the child’s gut microbiome composition at 5 years of age (Fig. 8). Prior studies have mostly focused on the effect of GDM on infant microbiome composition, with few studies in children older than 3 years of age (71–73). Previous results from the source population (PREWICE II) showed a lower frequency of whole grain intake and lower overall diet quality among women who developed GDM compared with non-GDM women (23), which could partly explain the finding in the present study. It can therefore not be excluded that shared dietary habits between mothers and their 5-year-old children may have contributed to the observed associations, although evidence from a recent systematic review indicates that such a parent–child dietary resemblance is generally weak to moderate (74).

Although cesarean section delivery is often linked to altered early microbial colonization, frequently delaying the establishment of a diverse and resilient microbiome (75, 76), delivery mode explained 7.6% of the gut microbiome composition 4–6 weeks after the introduction of solid foods (B2) but not at other time points (Fig. 8). Breast milk has a known role in shaping early microbial profiles, particularly *Bifidobacterium* dominance (77). In the IceGut cohort, differences in Jaccard distribution between breastfed and non-breastfed infants were detected 4–6 weeks after the introduction of solid foods (B2) and at 1 year of age (B3), when 73.5% and 32.4%, respectively, of children were still breastfed to some extent (Table S3; Fig. 5c and d). Comparisons at time point B1 were not possible due to the high prevalence of breastfeeding, with 99.3% of infants receiving breast milk before the introduction of solid foods (B1) (Table S3). Those who were still breastfed at the time of B2 and B3 sampling exhibited lower Shannon alpha diversity, suggesting that the gut microbiome had not yet fully transitioned to the composition typically associated with exclusive solid food intake (Fig. S2c and d).

Archaea were detected in 40.7% of maternal samples, with an average relative abundance of 0.2%. Although not numerically dominant, the presence of methanogenic archaea supports a potential role in gut ecosystem stability by contributing to hydrogen removal and influencing fermentation stability (78, 79). At 2 years of age (B4), 5 years of age (B5), and in maternal samples (M), individuals positive for archaea exhibited significantly higher alpha diversity compared with those without archaeal detection (Fig. S2a and b; Fig. 3c). This presence, coupled with significantly higher alpha diversity metrics, underscores their potential contribution to the maternal gut microbiome and the establishment of a more diverse microbial community (78, 79).

A progressive increase in the relative abundance of methanogenic archaea with age was evident in the children, particularly *Methanobrevibacter*, which was not detected before the introduction of solid foods and reached a prevalence of 18% by age 5 (data not shown). *Methanosphaera* was also detected in a few samples from the children, but at much lower frequencies. This concurrent increase in archaeal prevalence and age, especially of *Methanobrevibacter* (Table S4), paralleled the increase in alpha diversity across time points (Fig. 4a) and supports the findings from a Swedish study, suggesting that *Methanobrevibacter* has not yet reached adult levels by the age of 5 but continues to increase alongside rising Shannon alpha diversity (35). Although primers designed to target the bacterial V4 region of the 16S rRNA gene were used in the present study, methanogenic archaea were successfully detected. This is consistent with prior work by Pausan et al. (80), who noted that while archaeal-specific primers recover greater archaeal diversity, V4-targeting primers still detect dominant methanogens such as *Methanobrevibacter* in human stool samples. Nevertheless, future studies should validate these findings by employing archaea-specific primers or quantitative methods such as targeted qPCR to better quantify and assess the abundance and ecological relevance of these low-abundance taxa.

Overall, this study charts the growth of the gut microbiome development from birth to 5 years, identifying major shifts around the introduction of solid foods, a near-adult profile by 2 years, and later refinements at 5 years while also providing a profile of the maternal gut microbiome. These patterns show that longitudinal follow-up, rather than a single time point snapshot, is essential to determining when changes occur and distinguishing transient from persistent associations.

### Conclusion

This study provides the first description of gut microbiome development in Icelandic children from birth to 5 years of age, alongside an analysis of the maternal microbiome postpartum. The infant community moved from an early *Bifidobacterium*-dominated profile toward an adult-like configuration by 2 years, with 1 year marking a clear change in both alpha and beta diversities. By 5 years, children had higher observed richness than mothers, whereas mothers retained greater evenness, consistent with partial postpartum recovery. The relative abundance of *Blautia* appeared to increase at the 1-year age point, earlier than in comparable studies. Samples positive for archaea at 2 years, 5 years, and in mothers showed significantly higher alpha diversity compared with archaea-negative samples. Although overall microbial community composition became increasingly adult-like by 2 years of age, subtle differences persisted through age 5. Association with pre- and post-natal factors, particularly GDM, showed no significant microbial association until 5 years of age, which might indicate that some associations may only become apparent in later childhood, emphasizing the necessity of long-term studies.

## Data Availability

Sequencing data are deposited in National Center for Biotechnology Information (NCBI’s) Sequence Read Archive with the BioProject number PRJNA1256373.

## SUPPLEMENTAL MATERIAL

Supplemental Figures msphere.00745-25-s0001.pdf Fig. S1 and S2.

Supplemental Tables msphere.00745-25-s0002.pdf Tables S1 to S6.

ASM does not own the copyrights to Supplemental Material that may be linked to, or accessed through, an article. The authors have granted ASM a non-exclusive, world-wide license to publish the Supplemental Material files. Please contact the corresponding author directly for reuse.
