# Supplementary material for: Results from the IceGut study: tracking the gut microbiome development from mothers and infants up to five years of age
Source: mSphere. 2025 Dec 2;10(12):e00745-25. doi: 10.1128/msphere.00745-25 (PMC12724365; doi:10.1128/msphere.00745-25)
Supplement: Supplemental Figures — Fig. S1 and S2. [file msphere.00745-25-s0001.pdf]

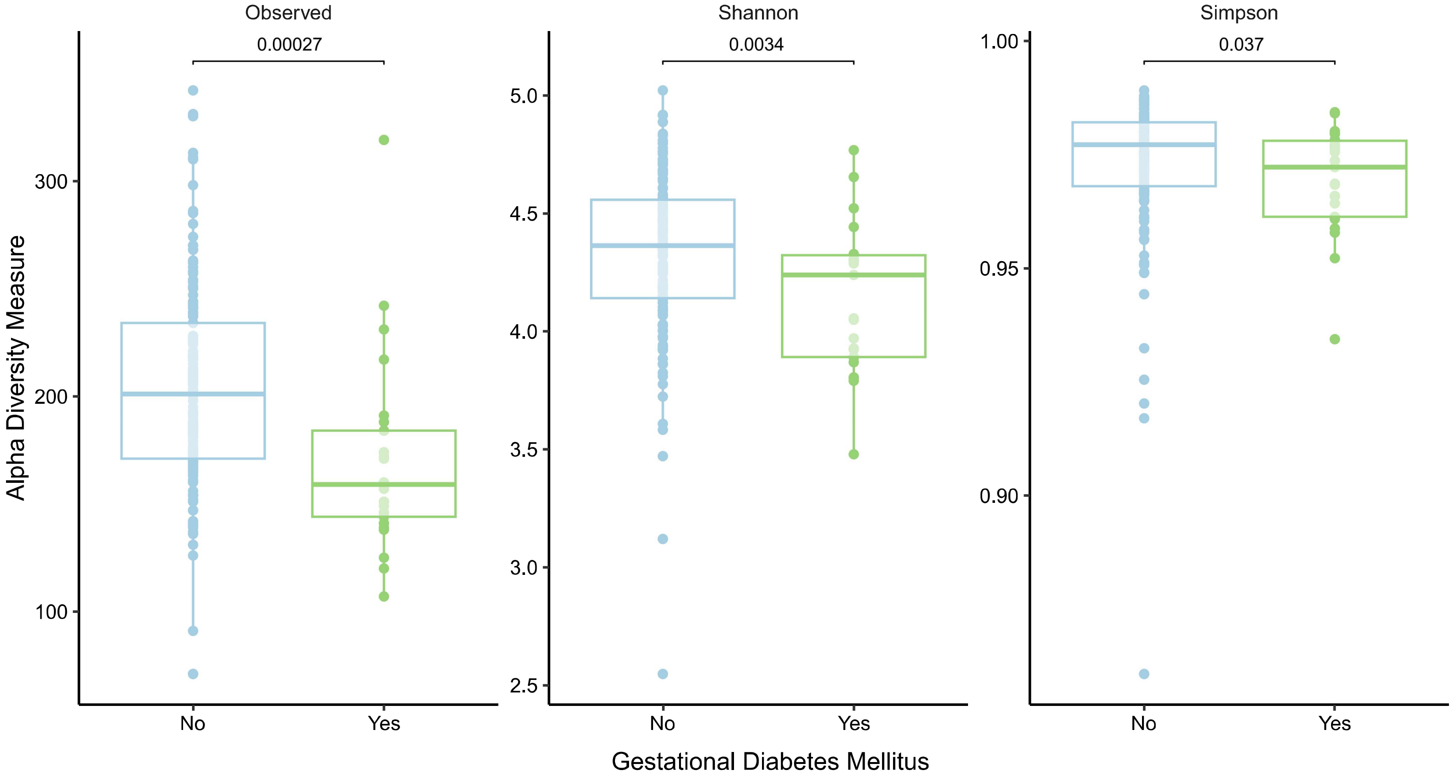

Fig S1. Alpha diversity indices (Observed, Shannon and Simpson) of mothers with and without GDM.

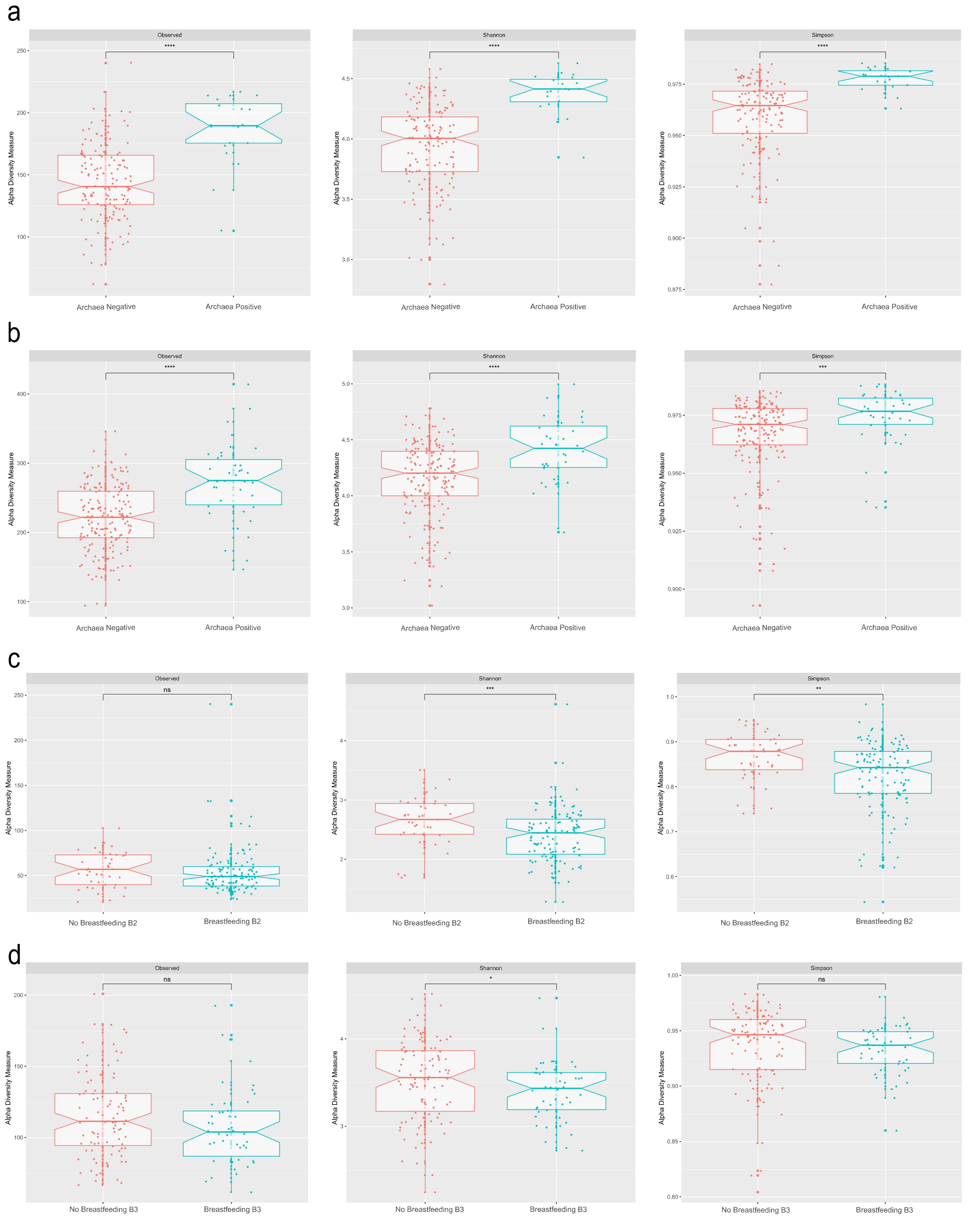

Fig S2. Comparison of alpha diversity indices (Observed, Shannon and Simpson) between archaea-positive and archaea-negative children at **(a)** two years of age and **(b)** five years of age. Comparisons of alpha diversity indicies (Observed, Shannon and Simpson) between children who were breastfeed and not **(c)** 4-6 weeks after introduction of solid foods and **(d)** at one years of age. Boxplots show median values with interquartile ranges, and individual points represent samples. Statistical differences between groups were assessed using the Wilcoxon rank-sum test. Significance levels are indicated as follows: ns = not significant ($p > 0.05$), * = $p < 0.05$, ** = $p < 0.01$, *** = $p < 0.001$, and **** = $p < 0.0001$.
