# Supplementary material for: Results from the IceGut study: tracking the gut microbiome development from mothers and infants up to five years of age
Source: mSphere. 2025 Dec 2;10(12):e00745-25. doi: 10.1128/msphere.00745-25 (PMC12724365; doi:10.1128/msphere.00745-25)
Supplement: Supplemental Tables — Tables S1 to S6. [file msphere.00745-25-s0002.pdf]

Table S1: Weekly food intake reported in food frequency questionnaire (FFQ) administered at gestational weeks 11 - 14. Values are expressed as reported frequencies per week*. Each column corresponds to a different sampling time point and complete series (referring to cases where all five child samples, and one maternal sample were provided).

	**ALL**	**B1**	**B2**	**B3**	**B4**	**B5**	**M**	**Complete series**	**p value**
	**328**	**157**	**266**	**153**	**162**	**213**	**214**	**43**	
**FFQ, frequency per week**	**Median (10th-90th percentile)**								
Fruits and vegetables	21.0 [10.0, 28.0]	21.0 [10.0, 28.0]	21.0 [10.0, 28.0]	19.0 [10.0, 28.0]	20.0 [10.0, 28.0]	16.5 [10.0, 28.0]	21.0 [10.0, 28.0]	16.5 [10.0, 32.0]	0.899
Fish, lean	1.0 [0.5, 1.8]	1.0 [0.5, 2.5]	1.0 [0.5, 1.0]	1.0 [0.5, 2.5]	1.0 [0.5, 2.5]	1.0 [0.5, 1.0]	1.0 [0.5, 2.5]	1.0 [0.5, 2.5]	0.984
Fish, fatty	0.3 [0.1, 1.0]	0.3 [0.1, 1.0]	0.5 [0.1, 1.0]	0.3 [0.1, 0.5]	0.5 [0.1, 1.0]	0.3 [0.1, 1.0]	0.5 [0.1, 1.0]	0.5 [0.2, 1.0]	0.974
Red meat	1.0 [0.5, 2.5]	1.0 [0.5, 2.5]	1.0 [0.5, 2.5]	1.0 [0.5, 2.5]	1.0 [0.5, 2.5]	1.0 [0.5, 2.5]	1.0 [0.5, 2.5]	2.5 [0.5, 2.5]	0.873
Poultry	1.0 [0.5, 2.5]	1.0 [1.0, 2.5]	1.0 [0.5, 2.5]	1.0 [0.5, 2.5]	1.0 [0.5, 2.5]	1.0 [0.5, 2.5]	1.0 [0.5, 2.5]	1.0 [1.0, 2.5]	0.97
Processed meat	0.5 [0.1, 1.0]	0.5 [0.3, 1.0]	0.5 [0.1, 1.0]	0.5 [0.1, 1.0]	0.5 [0.1, 1.0]	0.5 [0.1, 1.0]	0.5 [0.3, 1.0]	0.5 [0.3, 1.0]	0.996
Beans, nuts and seeds	1.0 [0.3, 2.5]	1.0 [0.3, 2.5]	1.0 [0.3, 2.5]	0.5 [0.3, 2.5]	1.0 [0.3, 2.5]	1.0 [0.3, 2.5]	1.0 [0.3, 2.5]	1.0 [0.5, 2.5]	0.924
French fries and chips	0.5 [0.5, 1.0]	0.5 [0.5, 1.0]	0.50 [0.5, 1.0]	0.5 [0.3, 1.0]	0.5 [0.5, 1.0]	0.5 [0.5, 1.0]	0.5 [0.5, 1.0]	1.0 [0.5, 1.0]	0.842
Vegetable oil for cooking	5.0 [2.5, 7.0]	5.0 [2.5, 7.0]	5.0 [2.5, 7.0]	5.0 [2.5, 7.0]	5.0 [2.5, 7.0]	5.0 [2.5, 7.0]	5.0 [2.5, 7.0]	5.0 [2.5, 7.0]	0.881
Butter for cooking	1.0 [0.5, 2.5]	1.0 [0.5, 2.5]	1.0 [0.5, 2.5]	1.0 [0.5, 2.5]	1.0 [0.5, 2.5]	1.0 [0.5, 2.5]	1.0 [0.5, 2.5]	1.0 [0.5, 2.5]	0.996
Butter on bread	5.0 [2.5, 7.0]	5.0 [2.5, 7.0]	5.0 [2.5, 7.0]	5.0 [2.5, 7.0]	5.0 [2.5, 7.0]	5.0 [2.5, 7.0]	5.0 [2.5, 7.0]	5.0 [2.5, 7.0]	0.961
Sour dairy	2.5 [1.0, 5.0]	2.5 [1.0, 5.0]	2.5 [1.0, 5.0]	2.5 [1.0, 7.0]	2.5 [1.0, 5.0]	2.5 [1.0, 5.0]	2.5 [1.0, 5.0]	2.5 [1.0, 5.0]	0.965
Cheese	5.0 [2.5, 7.0]	5.0 [4.4, 7.0]	7.0 [5.0, 7.0]	7.0 [5.0, 7.0]	6.0 [2.5, 7.0]	5.0 [2.5, 7.0]	5.0 [5.0, 7.0]	7.0 [5.0, 7.0]	0.462
Whole milk	0.1 [0.1, 1.0]	0.1 [0.1, 1.0]	0.1 [0.1, 1.0]	0.1 [0.1, 1.0]	0.1 [0.1, 1.0]	0.1 [0.1, 1.0]	0.1 [0.1, 1.0]	0.3 [0.1, 3.8]	0.171
Low fat milk	0.2 [0.2, 2.6]	0.2 [0.2, 2.6]	0.2 [0.2, 2.6]	0.2 [0.2, 1.1]	0.2 [0.2, 1.8]	0.2 [0.2, 2.6]	0.2 [0.2, 2.5]	0.2 [0.2, 1.6]	0.977
Skimmed milk	0.2 [0.2, 0.2]	0.2 [0.2, 0.2]	0.2 [0.2, 0.2]	0.2 [0.2, 0.2]	0.2 [0.2, 0.2]	0.2 [0.2, 0.2]	0.2 [0.2, 0.2]	0.2 [0.2, 0.2]	0.879
Coffee	0.5 [0.1, 7.0]	0.5 [0.1, 7.0]	0.5 [0.1, 7.0]	0.3 [0.1, 7.0]	0.1 [0.1, 5.0]	0.1 [0.1, 7.0]	0.5 [0.1, 7.0]	2.5 [0.1, 7.0]	0.653
Wholegrains	3.0 [1.4, 4.8]	3.7 [1.3, 4.8]	3.6 [1.4, 4.8]	3.0 [1.4, 4.8]	3.0 [1.3, 4.8]	3.6 [1.4, 5.0]	3.6 [1.4, 4.8]	3.8 [1.4, 4.9]	0.99
White bread	2.5 [0.5,2.5]	1.0 [0.5, 2.5]	2.5 [0.5, 2.5]	2.5 [0.5, 5.0]	2.5 [0.5, 2.5]	2.5 [0.5, 2.5]	2.5 [0.5, 2.5]	2.5 [0.5, 2.5]	0.871
Cakes, sweets, ice cream and cookies	3.5 [1.5, 6.0]	3.0 [1.5, 5.3]	3.5 [1.5, 6.0]	3.5 [1.5, 6.0]	3.5 [1.5, 6.0]	3.5 [1.5, 6.0]	3.5 [1.5, 6.0]	3.5 [2.0, 5.3]	0.993
Fruit juice	1.0 [0.5, 2.5]	1.0 [0.5, 2.5]	1.0 [0.5, 2.5]	1.0 [0.3, 2.5]	1.0 [0.5, 2.5]	1.0 [0.5, 2.5]	1.0 [0.5, 2.5]	1.0 [0.4, 2.5]	0.992
Soft drinks	1.1 [0.6, 3.1]	1.1 [0.6, 3.0]	1.1 [0.6, 3.0]	1.1 [0.6, 2.6]	1.1 [0.4, 2.6]	1.1 [0.6, 3.0]	1.1 [0.6, 3.0]	1.1 [0.6, 2.6]	0.552
Total milk products[a]	13.6 [8.2, 18.9]	14.5 [8.4, 18.3]	13.7 [8.2, 19.6]	14.5 [8.5, 20.5]	13.6 [8.0, 19.8]	14.0 [8.4, 19.1]	14.5 [8.3, 19.8]	14.5 [9.6, 22.6]	0.937

*Data are presented as medians and percentiles (10th-90th).

[a]Total milk products include sour dairy, cheese, whole milk, fat free milk and skimmed milk

p-value claculated with Kruskal-Wallis test

Table S2: Antibiotic use before each sampling time point for mothers and their children.

	B1	B2	B3	B4	B5	M
**B1 antibiotic use (since birth)**						
No	113 (96.6%)					
Yes	4 (3.4%)					
Missing	40					
**B2 antibiotic use (since birth)**						
No		127 (83.6%)				
Yes		25 (16.4%)				
Missing		114				
**B3 antibiotic use (last six months)**						
No			96 (65.8%)			
Yes			49 (33.6%)			
Don't know			1 (0.7%)			
Missing			7			
**B4 antibiotic use (last twelve months)**						
No				70 (50.7%)		
Yes				66 (47.8%)		
Don't know				2 (1.5%)		
Missing				24		
**B5 antibiotic use (last twelve months)**						
No					165 (80.9%)	
Yes					25 (12.3%)	
Don't know					8 (3.9%)	
Don't want to answer					6 (2.9%)	
Missing					9	
**Mothers' antibiotic use[a]**						
No						83 (53.2%)
Yes						61 (39.1%)
Don't know						12 (7.7%)
Missing						58

[a] Antibiotic use during pregnancy, at birth, or prior to mother's fecal sample collection.

Table S3

a) Feeding status at the time of initial sample collection (B1).

	**Total cohort**	**Complete series**
	**B1**	**B1**
N	157	46
Missing information	46	9
Exclusively breastfed (%)	49.5	43.2
Mix fed (breast + formula feeding) (%)	49.5	56.8
Exclusively formula feeding (%)	1	0

b) Breastfeeding status at each sampling time point (B1-B5), including responses to breastfeeding questions, the prevalence of any breastfeeding, and the mean total breastfeeding duration as reported at B5.

	**B1**	**B2**	**B3**	**B4**	**B5**
Answered	133	161	146	160	
Missing information	24	58	7	2	
Any breast feeding (%)	99.30%	73.50%	32.20%	4.40%	0%
Average breastfeeding duration					10 - 12 months

Continuous variables are shown as median and interquartile range; Distributions in categorical variable are presented as percentages.

Table S4: Relative abundance of genera with mean values exceeding 1% in at least one group (B1, B2, B3, B4, B5 or mothers). Methanobrevibacter is also shown despite remaining below the 1% threshold in all groups.

**Genus**	**B1**	**B2**	**B3**	**B4**	**B5**	**M**
Bifidobacterium	31.1227	26.2616	11.6606	8.1582	6.9183	5.7012
Bacteroides	16.0152	16.8617	16.9208	13.8924	12.0732	12.6597
Escherichia-Shigella	9.4185	5.2371	1.4383	0.2772	0.0549	0.2371
Clostridium	7.4890	5.0370	2.0945	1.6288	1.5941	0.8017
[Ruminococcus] gnavus group	3.5885	4.8398	1.5405	0.3624	0.0581	0.1793
Veillonella	3.2492	4.3339	0.6577	0.0921	0.0158	0.0398
Parabacteroides	2.9605	1.8406	1.1826	1.0633	0.9958	1.2697
Collinsella	2.4083	1.9482	1.7228	1.4938	0.5660	2.0611
Akkermansia	2.3894	2.4434	1.3565	1.7534	0.7200	1.3120
Thomasclavelia	2.3283	1.6717	1.2570	0.4258	0.1915	0.2339
Lacticaseibacillus	2.1649	1.5343	0.1108	0.0940	0.0307	0.0129
Streptococcus	1.8676	2.1234	2.2719	1.4921	0.6562	0.5668
Unknown *Enterobacteriaceae*	1.2299	1.0114	0.2261	0.0327	0.0010	0.0114
Blautia	1.1704	4.7111	10.4862	11.4485	12.6395	10.7116
Enterococcus	1.0497	1.1387	0.1396	0.0394	0.0002	0.0208
Sutterella	0.6048	1.2972	1.0669	1.0773	0.7450	0.8785
Faecalibacterium	0.0217	1.2407	9.7489	8.8712	8.3917	6.7741
Anaerostipes	0.1147	1.1998	3.2633	2.8514	2.8255	2.0083
Mediterraneibacter	0.5717	1.1900	1.2843	1.4777	1.2622	1.3803
[Clostridium] innocuum group	0.5420	1.0299	0.4288	0.1050	0.0342	0.0664
Agathobacter	0.1646	0.3528	2.4533	2.8301	3.6905	3.0581
Ruminococcus	0.0065	0.1078	1.8870	2.9828	3.1073	3.0955
Fusicatenibacter	0.0402	0.8349	1.6778	1.9303	1.9655	2.1241
Roseburia	0.0102	0.2989	1.4579	1.2279	1.3653	1.2423
Segatella	0.0089	0.1280	1.3211	3.7591	3.8159	1.7028
Alistipes	0.4139	0.2502	1.2993	2.2677	2.4372	2.6626
Erysipelotrichaceae UCG-003	0.0217	0.1835	1.2209	1.4513	1.6222	1.6745
Gemmiger	0.0232	0.2587	0.9383	1.6679	2.5282	2.1684
Anaerobutyricum	0.0008	0.1212	0.8573	1.1839	0.7325	1.1472
Lachnospiraceae NK4A136 group	0.3329	0.1738	0.8804	1.1706	0.9421	0.8667
Coprococcus	0.0130	0.0486	0.4993	1.1408	1.3385	1.5572
Christensenellaceae R-7 group	0.0010	0.0100	0.2866	0.8274	1.9861	1.8495
UCG-002	0.0067	0.0186	0.2063	0.7633	1.9368	1.7990
Unknown *Lachnospiraceae*	0.2755	0.6533	0.7279	0.8614	1.2938	0.9963
CAG-352	0.0013	0.0283	0.4094	0.9095	1.1392	0.9389
Dorea	0.0440	0.1851	0.6113	0.9330	1.0335	1.2158
Clostridia UCG-014	0.0004	0.0271	0.1940	0.3288	0.6960	1.1325
Methanobrevibacter	0.0000	0.0004	0.0016	0.0660	0.0408	0.2242
Other *archea*	0.0000	0.0000	0.0001	0.0005	0.0014	0.0120
Other	8.3279	9.3678	14.2120	17.0608	18.5533	23.6057

Table S5: Relative abundance of phyla with mean values exceeding 1% in at least one group (B1, B2, B3, B4, B5 or M). Archaeal taxa are also shown despite remaining below the 1% threshold in all groups.

Phyla	B1	B2	B3	B4	B5	M
Firmicutes	29.725541	40.273052	58.156364	62.413655	67.845325	65.563221
Bacteroidetes	19.600054	19.312596	21.813029	23.046802	21.792959	21.285484
Actinobacteria	35.179515	28.818995	13.715987	9.974908	7.728624	8.296942
Proteobacteria	12.941494	8.962893	4.663539	2.291550	1.431066	2.432286
Verrucomicrobia	2.389509	2.443839	1.360111	1.773901	0.772584	1.376176
Archaea	0.000040	0.000351	0.001749	0.066605	0.042230	0.236264
Other	0.163848	0.188275	0.289222	0.432579	0.387212	0.809627

Table S6: List of pathways showing statistically significant differences between groups based on functional gene count prediction.

	GeneSet	AdjPval	desc
59	M00001	0	Glycolysis (Embden-Meyerhof pathway), glucose => pyruvate
13	M00002	0	Glycolysis, core module involving three-carbon compounds
46	M00003	0	Gluconeogenesis, oxaloacetate => fructose-6P
21	M00004	0	Pentose phosphate pathway (Pentose phosphate cycle)
39	M00009	0	Citrate cycle (TCA cycle, Krebs cycle)
44	M00011	0	Citrate cycle, second carbon oxidation, 2-oxoglutarate => oxaloacetate
5	M00016	0	Lysine biosynthesis, succinyl-DAP pathway, aspartate => lysine
38	M00017	0	Methionine biosynthesis, aspartate => homoserine => methionine
22	M00018	0	Threonine biosynthesis, aspartate => homoserine => threonine
4	M00022	0	Shikimate pathway, phosphoenolpyruvate + erythrose-4P => chorismate
19	M00023	0	Tryptophan biosynthesis, chorismate => tryptophan
55	M00024	0	Phenylalanine biosynthesis, chorismate => phenylpyruvate => phenylalanine
32	M00026	0	Histidine biosynthesis, PRPP => histidine
2	M00034	0	Methionine salvage pathway
58	M00036	0	Leucine degradation, leucine => acetoacetate + acetyl-CoA
24	M00048	0	De novo purine biosynthesis, PRPP + glutamine => IMP
8	M00051	0	De novo pyrimidine biosynthesis, glutamine (+ PRPP) => UMP
6	M00060	0	KDO2-lipid A biosynthesis, Raetz pathway, LpxL-LpxM type
47	M00082	0	Fatty acid biosynthesis, initiation
31	M00083	0	Fatty acid biosynthesis, elongation
40	M00087	5.66E-293	beta-Oxidation
35	M00117	1.78E-144	Ubiquinone biosynthesis, prokaryotes, chorismate (+ polyprenyl-PP) => ubiquinol
49	M00120	0	Coenzyme A biosynthesis, pantothenate => CoA
52	M00121	0	Heme biosynthesis, plants and bacteria, glutamate => heme
48	M00126	0	Tetrahydrofolate biosynthesis, GTP => THF
9	M00144	0	NADH:quinone oxidoreductase, prokaryotes
43	M00145	1.80E-18	NAD(P)H:quinone oxidoreductase, chloroplasts and cyanobacteria
33	M00151	6.66346336546089e-320	Cytochrome bc1 complex respiratory unit
45	M00165	0	Reductive pentose phosphate cycle (Calvin cycle)
50	M00173	0	Reductive citrate cycle (Arnon-Buchanan cycle)
27	M00307	0	Pyruvate oxidation, pyruvate => acetyl-CoA
26	M00346	0	Formaldehyde assimilation, serine pathway
34	M00356	0	Methanogenesis, methanol => methane
37	M00357	0	Methanogenesis, acetate => methane
18	M00374	0	Dicarboxylate-hydroxybutyrate cycle
16	M00376	0	3-Hydroxypropionate bi-cycle
53	M00377	0	Reductive acetyl-CoA pathway (Wood-Ljungdahl pathway)
12	M00527	0	Lysine biosynthesis, DAP aminotransferase pathway, aspartate => lysine
17	M00529	0	Denitrification, nitrate => nitrogen
54	M00542	0	EHEC/EPEC pathogenicity signature, T3SS and effectors
36	M00545	0	Trans-cinnamate degradation, trans-cinnamate => acetyl-CoA
3	M00546	6.40E-93	Purine degradation, xanthine => urea
20	M00563	0	Methanogenesis, methylamine/dimethylamine/trimethylamine => methane
57	M00567	0	Methanogenesis, CO2 => methane
56	M00569	0	Catechol meta-cleavage, catechol => acetyl-CoA / 4-methylcatechol => propanoyl-CoA
41	M00620	0	Incomplete reductive citrate cycle, acetyl-CoA => oxoglutarate
42	M00651	0	Vancomycin resistance, D-Ala-D-Lac type
51	M00846	0	Siroheme biosynthesis, glutamyl-tRNA => siroheme
30	M00866	0	KDO2-lipid A biosynthesis, Raetz pathway, non-LpxL-LpxM type
29	M00878	0	Phenylacetate degradation, phenylaxetate => acetyl-CoA/succinyl-CoA
7	M00912	0	NAD biosynthesis, tryptophan => quinolinate => NAD
11	M00924	0	Cobalamin biosynthesis, anaerobic, uroporphyrinogen III => sirohydrochlorin => cobyrinate a,c-diamide
1	M00925	0	Cobalamin biosynthesis, aerobic, uroporphyrinogen III => precorrin 2 => cobyrinate a,c-diamide
28	M00926	0	Heme biosynthesis, bacteria, glutamyl-tRNA => coproporphyrin III => heme
25	M00957	0	Lysine degradation, bacteria, L-lysine => glutarate => succinate/acetyl-CoA
10	M00958	0	Adenine ribonucleotide degradation, AMP => Urate
23	M00959	0	Guanine ribonucleotide degradation, GMP => Urate
15	M00975	0	Betaine degradation, bacteria, betaine => pyruvate
14	M00982	0	Methylcitrate cycle
